# Nemonoxacin pharmacokinetics/pharmacodynamics against *Escherichia coli* in an *in vitro* dynamic urinary tract infection model

**DOI:** 10.1128/aac.01368-25

**Published:** 2026-06-11

**Authors:** Chunye Qi, Ruohao Zhang, Xingyi Qu, Xiaofen Liu, Xingchen Bian, Fengjia Zhu, Jing Chen, Xin Li, Jing Zhang

**Affiliations:** 1 Institute of Antibiotics, Huashan Hospital, Fudan University, Shanghai, China; 2 Clinical Pharmacology Research Center, Huashan Hospital, Fudan University 12478, Shanghai, China; 3 Department of Clinical Pharmacy and Pharmacy Administration, School of Pharmacy, Fudan University 12478, Shanghai, China; 4 Key Laboratory of Antibiotic Clinical Pharmacology of the National Health Commission, Shanghai, China; 5 Joint Laboratory of Hospital and Enterprise for Pathogen Diagnosis of Drug-Resistant Bacterial lnfections and lnnovative Drug R&D, Shanghai, China; 6 Professional Technical Service Platform for Comprehensive Preclinical Pharmacodynamic Evaluation and Clinical Translation of Antibacterial Agents, Shanghai, China; 7 Research and Development Center, Zhejiang Medicine Co., Ltd., Zhejiang, China; University of Houston, Houston, Texas, USA

**Keywords:** nemonoxacin, *in vitro*pharmacokinetics/pharmacodynamics modeling, urinary tract infections, *Escherichia coli*

## Abstract

Nemonoxacin, a novel non-fluorinated quinolone, is approved for community-acquired pneumonia, but its efficacy against uncomplicated urinary tract infections (uUTIs) caused by *Escherichia coli* (*E. coli*) is uncertain. We aimed to establish an *in vitro* uUTIs model simulating urinary concentrations to evaluate dosing regimens. An *in vitro* model was developed using cation-adjusted Mueller–Hinton broth (CAMHB) and synthetic human urine (SHU) to reproduce urinary exposure. The model was applied to 12 *E. coli* clinical isolates and ATCC 25922 (MICs <0.03 to >512 mg/L) over 24 h. The effects of medium type on MICs and bacterial growth were assessed. Pharmacokinetics/pharmacodynamics (PK/PD) relationships were described using a sigmoid *E*_max_ model, with change in bacterial density (Δlog_10_ CFU/mL) and the area under the bacterial kill curve (AUBKC) as efficacy endpoints. Monte Carlo simulation (MCS) estimated the probability of target attainment (PTA) and cumulative fraction of response (CFR) for 500 mg and 750 mg once-daily regimens. Nemonoxacin MICs were higher in SHU, and bacterial growth showed early inhibition. The AUC_0–24_/MIC ratio best correlated with antibacterial effect (*R*² = 0.951 in CAMHB; 0.982 in SHU). For a 1-log CFU reduction, AUC_0–24_/MIC targets were 46.5 in CAMHB and 199 in SHU. The 500 mg regimen achieved ≥90% PTA for isolates with MIC ≤ 16 mg/L (CFR 92.1%) using CAMHB-derived targets but only ≤8 mg/L (CFR 59.4%) in SHU. A 500 mg once-daily regimen of nemonoxacin appears to be a promising therapeutic option for the treatment of uUTIs, although further clinical studies are necessary to validate these findings.

## INTRODUCTION

Uncomplicated urinary tract infections (uUTIs), including acute cystitis and pyelonephritis, are among the most common outpatient infections. Symptoms such as painful urination, urgency, frequency, and fever greatly affect quality of life, and treatment aims to provide rapid relief while preventing systemic spread (1, 2). Most uUTIs are caused by *Escherichia coli* (*E. coli*), though *Klebsiella pneumoniae*, *Staphylococcus saprophyticus*, and *Enterococcus* spp. are also involved (3). The pathogen spectrum and antibiotic sensitivity form the basis for the choice of antibiotic.

Quinolones are widely used due to their strong activity against *E. coli in vitro* (4) and high urinary concentrations (5) and remain recommended as a second-line treatment for uUTIs (6). However, resistance has risen sharply, with nearly half of urinary *E. coli* isolates in the Asia-Pacific region resistant to levofloxacin or ciprofloxacin (7). In response to the alarming prevalence of resistance, clinicians have attempted high-dose, short-course treatment regimens to reduce the likelihood of further resistance development. Nonetheless, a recent clinical pharmacokinetics and pharmacodynamics (PK/PD) study has indicated that the predicted cumulative fractions of response (CFRs) for a target area under the curve (AUC)_0–24_/MIC (the area under the concentration–time curve over 24 h in steady-state divided by the minimum inhibitory concentration [MIC]) ratio of 125 for *E. coli* were only 84.25% and 86.00% for standard-dose (500 mg) and high-dose (750 mg) orally administered levofloxacin, respectively (8). A similar trend has been observed with high-dose ciprofloxacin, which has proven ineffective against *E. coli* isolates with MIC values exceeding 1 mg/L (9). These findings underscore the need for new treatment options.

Nemonoxacin, a novel non-fluorinated quinolone approved for community-acquired pneumonia (CAP), demonstrated potent activity against a broad range of gram-positive bacteria and comparable activity against gram-negative bacteria to levofloxacin (4). Furthermore, it is predominantly excreted unchanged in urine, with 60%–70% of the drug recovered intact within a 72-h period (10), achieving concentrations far higher than in plasma. Multiple studies have shown that high urine concentrations play a vital role in the inhibition of growth or the eradication of bacteria in the urine (11, 12) and antibiotics with high urine exposure may effectively eradicate bacteria that were determined to be resistant based on *in vitro* susceptibility testing (13, 14). Therefore, this pharmacokinetic profile of nemonoxacin suggests its strong potential for treating uUTIs.

Yet, pre-clinical PK/PD evidence in the urinary setting is lacking. Due to the significant inter-individual variability in urine concentrations, previous studies have rarely simulated urine concentrations *in vitro*, making it difficult to obtain PK/PD relationships in urine. Besides, cation-adjusted Mueller–Hinton broth (CAMHB) is unsuitable for reproducing the host suppression of bacterial growth in the urine environment, the phenotype characteristics of bacteria adapting to the urine environment, and the antibiotic bactericidal effects in urine. Therefore, exploring how to simulate the urine environment for researching bacterial growth or eradication better has become a challenge in the study of uUTIs.

Therefore, this study aims to (i) establish an *in vitro* PK/PD model to simulate the growth of *E. coli* in urine and the urinary pharmacokinetics of nemonoxacin, (ii) obtain the optimal PK/PD index and corresponding target values, and (iii) evaluate the efficacy of standard-dose (500 mg, qd) and high-dose (750 mg, qd) regimens in uUTIs, providing evidence for expanding its indications.

## MATERIALS AND METHODS

### Media and antibiotics

Luria-Bertani broth with agar (Sangon Biotech, China), cation-adjusted Mueller–Hinton broth (CAMHB) (BD, USA), pooled human urine, and synthetic human urine (SHU) were used. Human urine was collected and pooled from healthy male volunteers and filter sterilized (Ethics Committee approval: Project no. 2019-310-1). SHU was modified from previous studies (9, 15) with the addition of 0.1% vol/vol casamino acids (stock solution 20% wt/vol) and 0.1% (vol/vol) yeast extract (stock solution 10% wt/vol) to best match the growth of *E. coli* in urine (Table S1). SHU was adjusted to pH equal to 5.6, 6.0, and 6.5 and then was filtered and sterilized.

Nemonoxacin malate (lot E0/E1, containing 72.2% nemonoxacin) was provided by Zhejiang Medicine Co., Ltd (Zhejiang, China). The stock solution was prepared with sterile distilled water and stored at −80°C for a maximum of 2 months before use. Levofloxacin was bought from the National Institutes for Food and Drug Control (lot 130455-201106, containing 97.1% levofloxacin).

### Bacteria strains

A total of 12 clinical *E. coli* isolates were used in this work, among which 5 isolates were extended-spectrum β-lactamases (ESBL) producing *E. coli. E. coli* ATCC 25922 served as the control strain.

### MIC determination in CAMHB and SHU

First, the MICs of nemonoxacin were determined against *E. coli* ATCC 25922 and 12 clinical isolates using the standard broth microdilution (BMD) method, performed in triplicate and reported as median values, in accordance with Clinical and Laboratory Standards Institute (CLSI) guidelines (16). Next, to determine the MIC in SHU (MIC_SHU_), CAMHB was replaced with SHU at pH values of 5.6, 6.0, and 6.5, while all other steps remained unchanged. Levofloxacin MICs were measured in parallel as a control, with SHU tested at pH 5.6 and 6.5 only.

Urine pH data from 1,290 healthy volunteers at the Clinical Pharmacology Research Center of Huashan Hospital (2023) showed an average pH of 6.2 ± 0.5. Because nemonoxacin forms crystalline salts with malate, higher pH values markedly affect drug exposure. For this reason, media with pH above 6.5 were not included in this study.

### *In vitro* UTIs model

**Fig 1:**
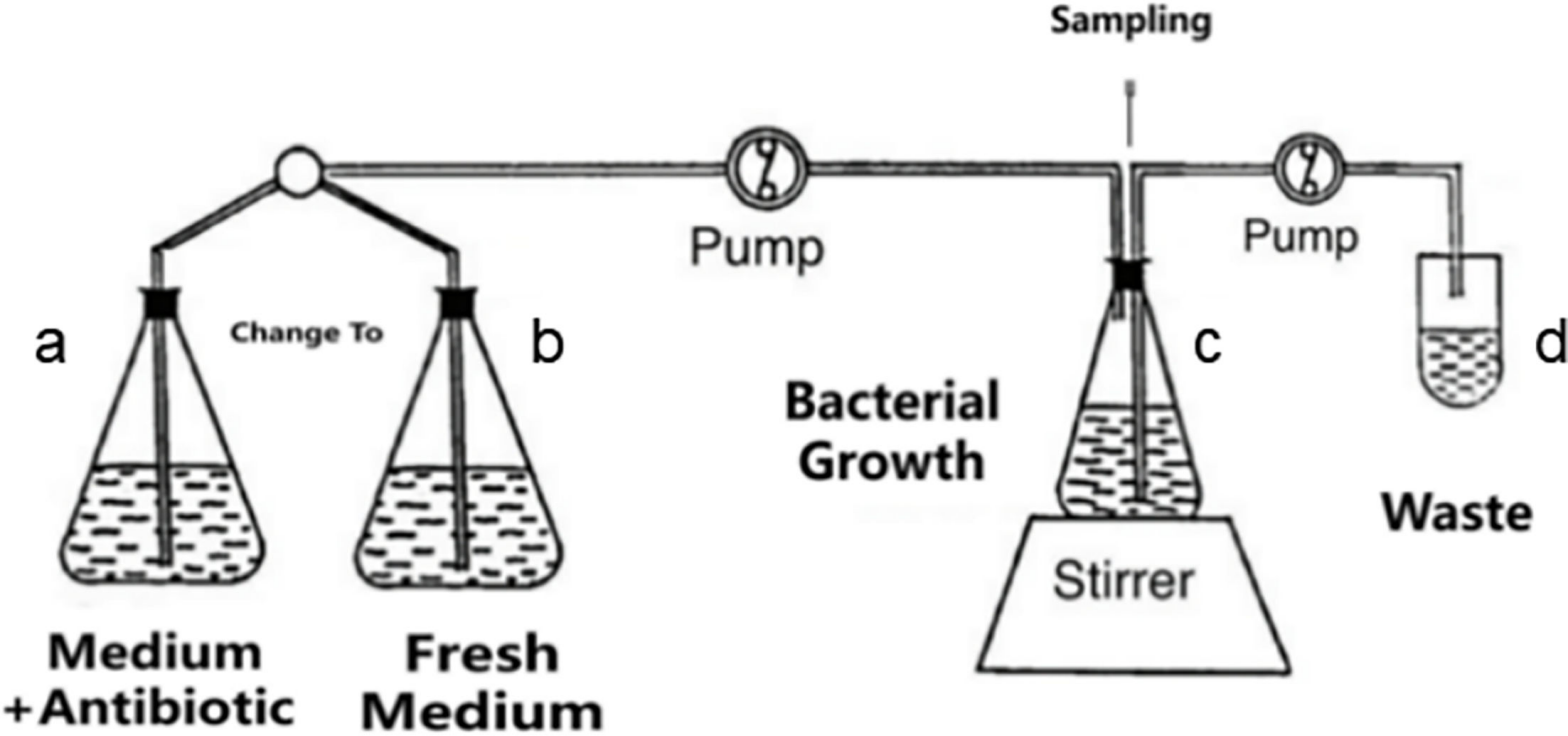
The schematic depiction of the *in vitro* dynamic UTI model set-up. Flask **a** represents a drug solution reservoir, **b** represents drug-free medium, **c** represents a central compartment containing bacterial suspension, and **d** represents a waste collection reservoir.

A one-compartment open dilution model was established using SHU (pH 6.5) as the medium to simulate urinary concentrations of nemonoxacin after oral dosing, based on the method of Satta et al. (Fig. 1) (17). The system consisted of a central compartment containing bacterial suspension (flask c) connected via silicone tubing to reservoirs for drug solution (flask a), drug-free medium (flask b), and waste (flask d). A peristaltic pump (Masterflex L/S; Cole-Parmer Co. Ltd.) regulated the inflow of drug or diluent to mimic absorption and clearance, while a magnetic stir bar ensured homogeneity.

Overnight bacterial cultures were inoculated at 1 × 10^6^ CFU/mL and preincubated at 35°C for 1 h before dosing (18). After that, different doses of nemonoxacin or sterile distilled water (control) were injected into the flask a, and the peristaltic pump was turned on. This system was adjusted so that in the absorption phase, the antibiotic solution in flask a was pumped into flask c until the antibiotic concentration reached the peak value. At this point, the system was switched from pumping the antibiotic solution to pumping the drug-free medium from flask b to flask c at a flow rate to mimic the in vivo clearance of nemonoxacin. Flow rates (F) were calculated to reflect observed PK profiles according to the following formula:


$$F=\frac{V_{C}}{\Delta t}\times \frac{C_{D_{i}}-C_{D_{i-1}}}{C_{D}-C_{D_{i-1}}}$$

where $C_{D_{i}}$ is the drug concentration at time i, $C_{D_{i-1}}$ is the drug concentration at time i-1, the volume of central glass compartment (*V*_*c*_) is assumed to be unchanged, and *C*_*D*_ is the drug concentration in the flask a (specific flow rates over the corresponding time intervals can be found in Table S2 in Supplemental material).

Pharmacokinetic simulations were based on steady-state urine concentrations in healthy Chinese volunteers receiving multiple oral doses of nemonoxacin (500 mg, once daily) (10). Urine samples collected at specified intervals (i.e., 0–2, 2–4, 4–8, 8–12, and 12–24 h) on day 10 were used to calculate median and minimum concentrations, representing high- and low-exposure groups (Table 1). Maximum concentrations were not simulated because the excretion rate significantly exceeded 100%. Simulated concentrations were validated against clinical data, with the slope of the linear regression equation ranging from 0.80 to 1.2.

**TABLE 1:** Statistical analysis of nemonoxacin urine concentrations (500 mg, qd, sample collected on day 10) over five time intervals

Sampling time intervals	Mean ± SD (mg/L)	Median^*a*^ (mg/L)	Minimum^*a*^ (mg/L)	Maximum (mg/L)
0–2 h	552.77 ± 387.57	535.34	150.76	1477.5
2–4 h	347.16 ± 345.04	176.6	99.88	1169.1
4–8 h	202.13 ± 90.43	201.81	50.49	315.14
8–12 h	160.82 ± 92.71	127.45	50.44	351.51
12–24 h	106.03 ± 50.36	90.16	48.11	214.62

^*a*^ Choose median and minimum urine exposure as high exposure and low exposure, respectively.

During PK/PD experiments, samples (0.6–0.8 mL) were collected with a syringe at 0, 0.5, 1, 2, 4, 6, 8, and 24 h, followed by centrifugation at 12,000 rpm for 6 min. The supernatant was stored at −40°C for subsequent determination of nemonoxacin concentration, and the pellet was washed, diluted, and plated for counting of viable bacteria.

Nemonoxacin concentrations in supernatants were determined using a validated liquid chromatography–tandem mass spectrometry (LC-MS/MS) method. Calibration was linear over the range of 0.020–10.00 mg/L, with a lower limit of quantification of 0.020 mg/L. Accuracy, precision, and selectivity met international bioanalytical validation standards (19, 20).

### Growth and time–kill curves of *E. coli* in different media

Bacterial growth was evaluated in drug-free CAMHB, SHU, and pooled human urine using two clinical isolates (W031-023 and W049-080) tested in duplicate. Samples were collected at 0, 0.5, 1, 2, 4, 6, 8, and 24 h to establish baseline growth, and bacterial density was measured at the same time points during 24-h nemonoxacin exposure. Samples were drawn from the central compartment, washed, centrifuged, and serially diluted. Afterward, 10 µL of each dilution was plated onto LB agar and incubated at 37°C for 18–24 h. Viable colonies were counted, expressed as CFU/mL, log-transformed, and plotted against time to generate time–kill curves.

### PK/PD index and targets

The PK data were analyzed by non-compartmental analysis (NCA) using Phoenix WinNonlin software (version 8.1, Certara Co. Ltd.). Subsequently, PK/PD indexes were calculated, including the peak concentration to MIC ratio (*C*_max_/MIC or *C*_max_/MIC_SHU_), the 24-h area under the concentration-time curve to MIC ratio (AUC_0–24_/MIC or AUC_0–24_/MIC_SHU_), and the cumulative percentage of 24 h that the drug concentration exceeds the MIC at steady-state conditions (%T > MIC or %T > MIC_SHU_). Protein binding in urine was negligible and thus not a factor in these calculations (9). A sigmoid *E*_max_ model was employed to characterize the relationship between the PK/PD indexes and the antibacterial effects, that is, changes in log_10_ CFU/mL between 24 and 0 h (Δlog_10_ CFU/mL) and the area under the bacterial kill curve (AUBKC). The formula can be described as follows:


$$E=E_{0}-\frac{E_{\max}\times X^{H}}{X^{H}+EC_{50}^{H}}$$

where *E* represents Δlog_10_ CFU or AUBKC, *E_0_* is the baseline value of the drug effect, *E*_max_ is the maximum drug effect *in vitro*, *X* represents the PK/PD index (*C*_max_/MIC, *C*_max_/MIC_SHU,_ AUC_0–24h_/MIC, AUC_0–24h_/MIC_SHU_, %T > MIC, or %T > MIC_SHU_), *EC*_50_ represents the value of PK/PD index when the drug effect reaches 50% of *E*_max_, and *H* represents the Hill coefficient.

The optimal PK/PD index that correlated with the efficacy of nemonoxacin was identified by comparing the correlation coefficient (*R*^2^). Following this, the PK/PD targets required for achieving net bacterial stasis (or EC_50_) and a 1-log_10_ CFU reduction from baseline (or EC_90_) were calculated.

### PTA and CFR analysis

The probability of target attainment (PTA) and the cumulative fraction of response (CFR) were evaluated for two nemonoxacin dosing regimens: 500 mg (standard dose) and 750 mg (high dose) administered once daily for 10 days. Monte Carlo simulations (MCS) were conducted using R software (version 4.3.3), generating PK data for 5,000 virtual patients with a normal random number generator. The PK/PD targets used for these calculations were derived from prior PK/PD analysis. All figures were plotted using GraphPad Prism (version 10.2.0).

## RESULTS

### MICs of nemonoxacin against *E. coli* strains in broth and SHU

**Fig 2:**
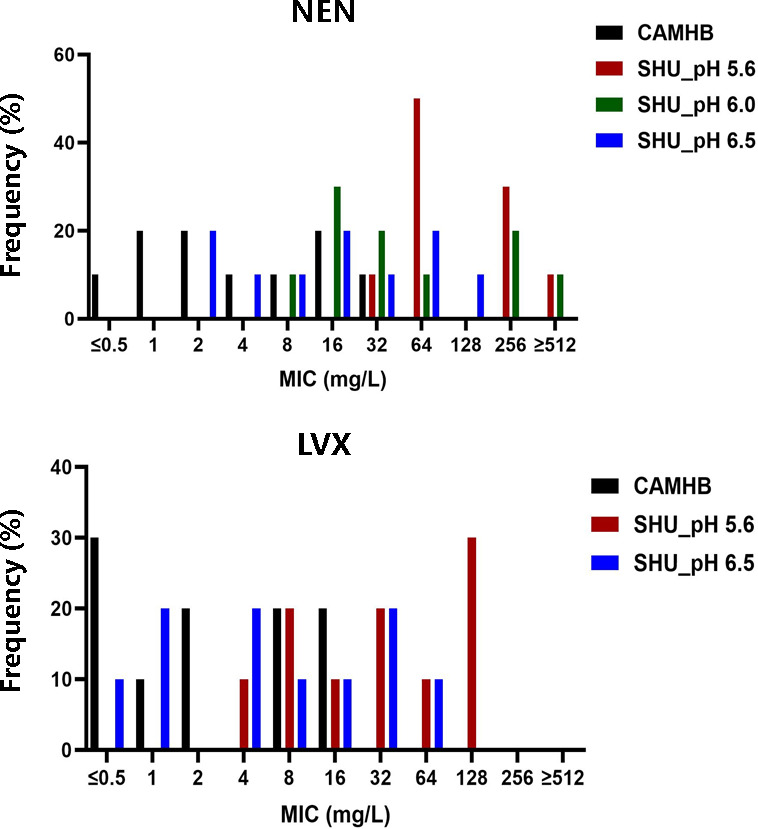
The MIC distribution of nemonoxacin and levofloxacin in CAMHB and SHU with different pH (5.6, 6.0, and 6.5 for nemonoxacin; 5.6 and 6.5 for levofloxacin).

Nemonoxacin MICs against 12 clinical *E. coli* isolates ranged from 0.5 to 128 mg/L; the MIC for the control strain ATCC 25922 was 0.03 mg/L (Fig. 2, detailed MIC values and ESBL type are in Table S3). MIC_SHU_s were markedly higher than MICs and were significantly affected by pH. Compared with standard testing in CAMHB, nemonoxacin MICs in SHU were higher by an average of 5 ± 2, 4 ± 2, and 2 ± 1-log_2_ dilutions at pH 5.6, 6.0, and 6.5, respectively.

### *E. coli* growth profiles in CAMHB, SHU, and pooled human urine

**Fig 3:**
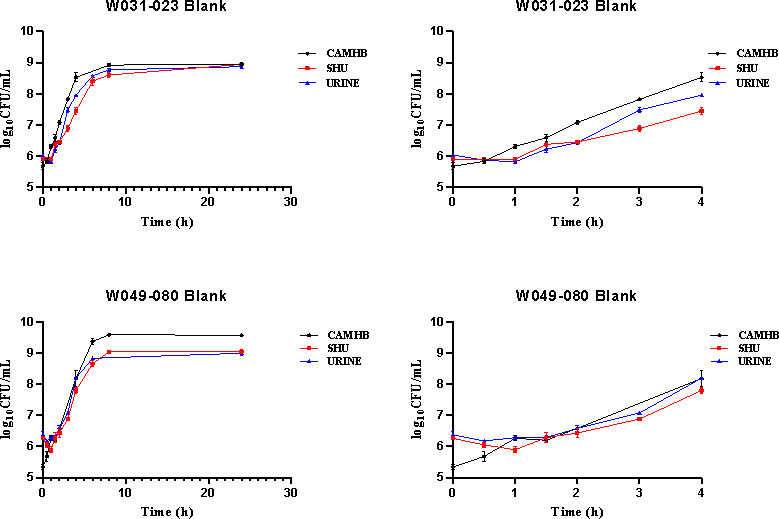
*E. coli* growth curves in CAMHB, SHU (pH = 6.5), and pooled human urine.

After a 1-h pre-culture, *E. coli* in pooled human urine and SHU showed an initial plateau from 0 to 2 h (Fig. 3), which was not observed in CAMHB.

Cultures in urine and SHU then entered logarithmic growth between 2 and 6 h before reaching a second plateau, whereas cultures in CAMHB grew logarithmically from the start until 8 h and then slowed to a plateau. The Δlog_10_ CFU/mL was 3.36 ± 0.08 in CAMHB, 2.91 ± 0.12 in SHU, and 2.72 ± 0.11 in pooled urine. Overall, SHU more closely reproduced the growth pattern observed in urine than CAMHB, supporting its use as a surrogate medium.

### PK validation of the dynamic *in vitro* model

**Fig 4:**
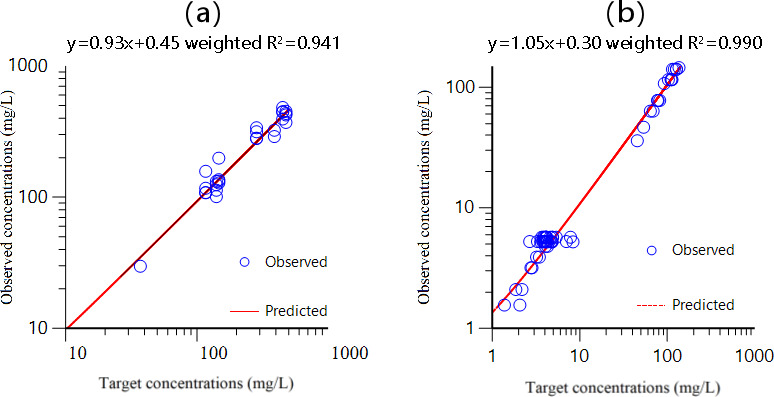
Accuracy of observed concentration of nemonoxacin in CAMHB (**a**) and SHU (**b**) vs. targeted concentration.

Figure 4 shows the linear fitting of the observed concentrations to the target concentrations of the *in vitro* PK model. In CAMHB, the linear fitting equation was *y* = 0.93x + 0.45 with a weighted *R*^2^ of 0.941. In SHU, the linear fitting equation was *y* = 1.05x + 0.30 with a weighted *R*^2^ of 0.990, suggesting that the PK profiles in the *in vitro* model can well match the PK profiles in human urine.

### Time–kill kinetics of nemonoxacin in the *in vitro* PK/PD model

**Fig 5:**
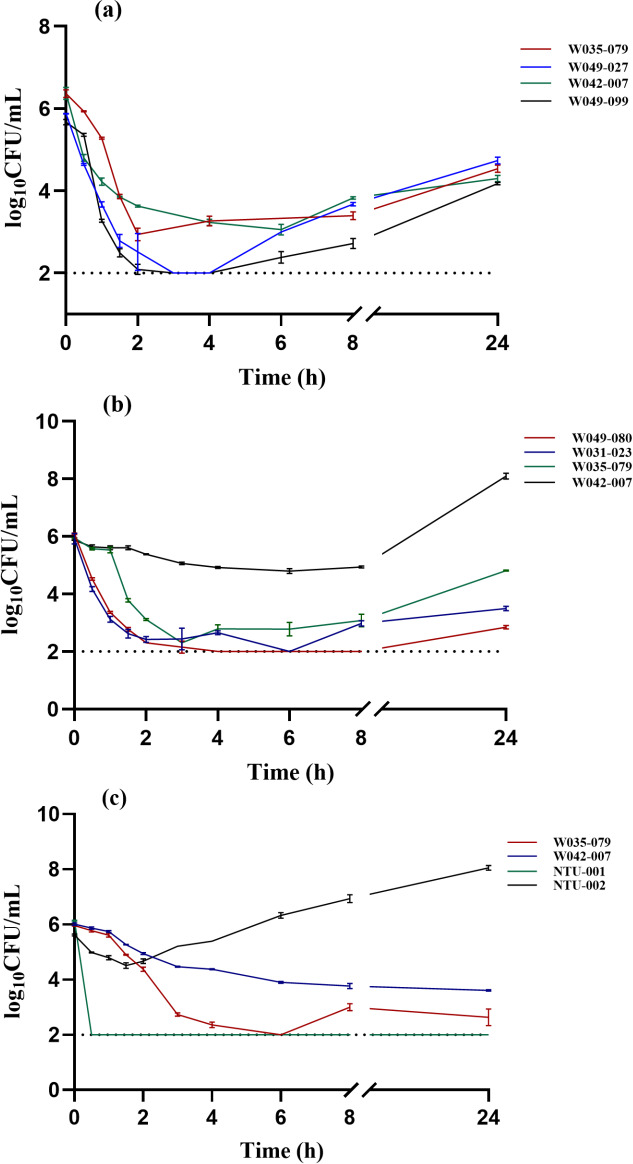
Time–kill curves of nemonoxacin low (**a and b**) and high (**c**) urine exposure groups against *E. coli* cultured in CAMHB (**a**) or SHU (**b and c**) (means ± SD; *n* = 2).

Under low urine exposure in CAMHB, nemonoxacin produced a rapid decline in bacterial counts across all tested *E. coli* strains (W035-079, W049-027, W042-007, and W049-099), with the largest reductions occurring within 4 h of dosing (Fig. 5). Regrowth emerged by 6 h, and the overall Δlog_10_ CFU/mL was ≤1, indicating primarily bacteriostatic activity in CAMHB under low urine exposure.

Notably, activity was slightly reduced in SHU under the same low exposure. Three strains (W049-080, W031-023, and W035-079) showed early declines within 4 h, followed by gradual regrowth after 8 h; the Δlog_10_ CFU/mL was ≤1, indicating the bacteriostatic activity in SHU. In contrast, strain W042-007 (MIC = 16 mg/L; MIC_SHU_ = 64 mg/L) showed only a slow initial decline, regrew from 6 h, and by 24 h was close to the control. Overall, our data suggested nemonoxacin of low urine exposure had bacteriostatic activity against *E. coli* in SHU.

With high urine exposure in SHU, three strains (W035-079, W042-007, and NTU-001) showed sustained reductions with Δlog_10_ CFU/mL ≤2, indicating bactericidal activity (Fig. 5). Strain NTU-002 (MIC = 128 mg/L; MIC_SHU_ = 512 mg/L) declined rapidly within 2 h but subsequently regrew, resulting in 24-h counts similar to control. Compared with low exposure, high exposure broadened activity to isolates with higher MICs (Table S3), though it remained insufficient to suppress all strains.

### PK/PD analysis

**Fig 6:**
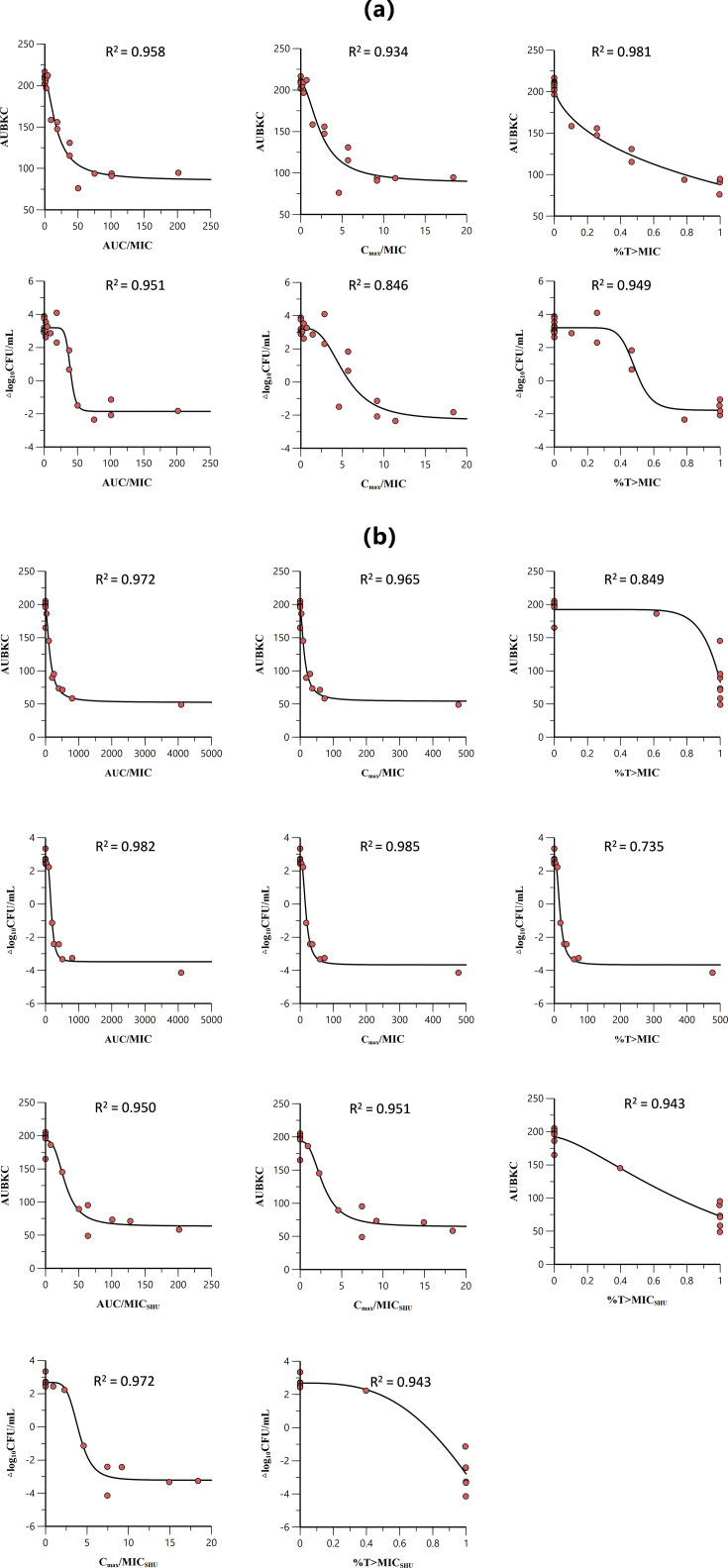
Correlation of PK/PD indexes with efficacy against *E. coli* for nemonoxacin in an *in vitro* UTI model for strains cultured in CAMHB (**a**) and SHU (**b**).

The sigmoid *E*_max_ model well described the exposure–response relationship for both endpoints (Δlog_10_ CFU/mL and AUBKC). Using Δlog_10_ CFU/mL as the endpoint, AUC_0–24_/MIC showed the strongest correlations with activity, with *R*^2^ values of 0.951 in CAMHB and 0.982 in SHU. Correlations with %T > MIC were weaker (*R*^2^ = 0.949 in CAMHB and 0.735 in SHU). Similar patterns were observed when using AUBKC (Fig. 6). Model parameters (*E*_0_, *E*_max_, EC_50_, and Hill coefficient) were reported in Table 2.

**TABLE 2:** Sigmoid *E*_max_ model fitting results and PK/PD targets for nemonoxacin against *E. coli* in CAMHB and SHU^*a*^

Media	PK/PD index	PD endpoint	*R* ^2^	*E*_max_	EC_50_	*E*_0_	*γ*	Stasis effect	Bactericidal effect		
CAMHB	*C*_max_/MIC	Δlog_10_ CFU	0.846	5.55	5.31	3.24	3.09				
		AUBKC	0.934	120	2.45	208	1.89				
	AUC_0–24h_/MIC	Δlog_10_ CFU	0.951	5.04	39.4	3.19	9.98	41.6	46.5 (−1log)		
		AUBKC	0.958	123	18.5	208	1.69	18.5	68.2 (−1log)		
	%T > MIC	Δlog_10_ CFU	0.949	4.97	0.487	3.19	10.0				
		AUBKC	0.981	426	4.79	207	0.604				
SHU	*C*_max_/MIC	Δlog_10_ CFU	0.985	6.42	17.4	2.75	2.55				
		AUBKC	0.965	140	12.9	194	1.67				
	AUC_**0–24h**_/MIC	Δlog_10_ CFU	0.982	6.20	177	2.72	3.50	165	199 (−1log)	246 (−2log)	358 (−3log)
		AUBKC	0.972	141	139	194	1.72	139	499		
	%T > MIC	Δlog_10_ CFU	0.735	66.6	1.29	2.70	9.95				
		AUBKC	0.849	1425	1.28	192	9.96				
	*C*_max_/MIC_SHU_	Δlog_10_ CFU	0.972	5.89	4.02	2.67	5.05				
		AUBKC	0.951	129	2.76	193	2.67				
	AUC_0–24h_/MIC_SHU_	AUBKC	0.950	129	29.9	193	2.87				
	%T > MIC_SHU_	Δlog_10_ CFU	0.943	32.2	1.68	2.68	3.05				
		AUBKC	0.943	254	1.09	192	1.48				

^*a*^ CAMHB, cation-adjusted Mueller−Hinton broth; SHU, synthetic human urine; *R^2^*, correlation coefficient; *E*_max_, the maximum drug effect *in vitro; *EC_50_*,* the value of PK/PD index when the drug effect reaches 50% of *E*_max_*; E*_0_ is the baseline value of the drug effect; H, the Hill coefficient; The targets reaching stasis effect are PK/PD values when Δlog_10 _CFU = 0 or EC = 50%; the targets reaching bacteriostatic effect are PK/PD values when Δlog_10 _CFU <-1 or EC = 90%.

From these models, the AUC_0–24_/MIC targets for stasis (0-log_10_ reduction or EC_50_) were 41.7 or 18.5 in CAMHB and 165 or 139 in SHU. For a 1-log_10_ reduction (or EC_90_), the targets were 46.5 or 68.2 in CAMHB and 199 or 499 in SHU, respectively (Table 2).

### PTA and CFR for two dosing regimens

**Fig 7:**
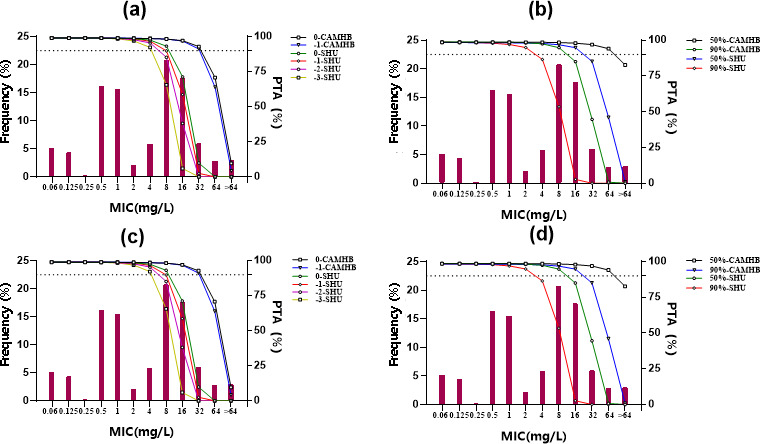
Probability of target attainment (PTA) of standard-dose (**a and b**) (500 mg, qd) and high-dose (**c and d**) (750 mg, qd) nemonoxacin based on different targets, overlaid on the MIC distribution for *E. coli*.

Using the Δlog_10_ CFU/mL-based targets (AUC_0–24_/MIC 46.5 in CAMHB; 199 in SHU), the 500 mg once-daily regimen achieved 90% PTA for isolates with MICs ≤ 32 mg/L (CAMHB target) or ≤ 8 mg/L (SHU target), yielding CFRs of 94.3% and 80.5%, respectively (Fig. 7). Using AUBKC-based targets (AUC_0–24_/MIC 68.2 in CAMHB; 499 in SHU), 90% PTA was achieved for MIC ≤ 16 mg/L or ≤ 2 mg/L, with CFRs of 92.1% and 59.36%, respectively. The 750 mg once-daily regimen produced similar PTA and CFR outcomes.

## DISCUSSION

Understanding drug concentrations at the site of infection is essential for maximizing bacterial killing. In the present work, we established a dynamic uUTI model that mimics human urodynamics to investigate the PK/PD relationships of nemonoxacin against *E. coli*. To the best of our knowledge, this is the first study to characterize the PK/PD of nemonoxacin in an *in vitro* dynamic PK/PD model based on human urinary exposure.

### Urine environment affects nemonoxacin antibacterial activity

Urine is unique from a microbial perspective, and its chemical makeup, distinct from all other bodily fluids, has been modeled for studying the growth of microbes for 50 years (21). Consistent with prior work (9, 15), we used SHU supplemented with casamino acids and yeast extract to reproduce *E. coli* growth seen in human urine while minimizing drug binding. In SHU, nemonoxacin’s activity decreased as acidity increased: MICs were higher than in CAMHB and rose further at lower pH. Similar pH-dependent effects were reported for ciprofloxacin MICs against *E. coli* determined in modified SHU medium and pooled human urine (9). Smith et al. (22) found that for quinolones containing piperidine groups, such as ciprofloxacin and ofloxacin, protonation of basic nitrogen atoms at acidic pH reduces their cell penetration and target binding, thus increasing MICs. Although nemonoxacin lacks a piperidine group, it still contains basic nitrogens, which likely accounts for its reduced activity in acidic urine.

### PK/PD target comparisons

A significant bacterial regrowth was observed in the time–kill curves. The selection of resistant subpopulations may explain this finding. Within 2 h, the susceptible bacterial population was rapidly eradicated, while resistant subpopulations may have survived, leading to regrowth at 24 h as nemonoxacin concentrations declined. Similar patterns were reported by Blaser et al. in an *in vitro* PK/PD study of enoxacin, where regrowth occurred within 24 h unless the *C*_max_/MIC ratio exceeded 8:1 (23).

According to our *in vitro* PK/PD study, AUC_0–24_/MIC was found to be the best predictor of microbiological efficacy for nemonoxacin, aligning with other quinolones (24, 25). Despite the PK data showing high nemonoxacin concentrations in urine, neither animal nor clinical PK/PD targets exist for UTIs. The UTI PK/PD target (AUC_0–24_/MIC of 46.9 for 1-log_10_ kill in 24 h) obtained in the present study is roughly equivalent to targets reported for *Streptococcus pneumoniae* (*S. pneumoniae*) in pneumonia patients, namely, AUC_0–24_/MIC of 63.3 and 93.7 for clinical efficacy and microbiological efficacy, respectively (26). Compared to ciprofloxacin and levofloxacin, nemonoxacin displays a relatively lower PK/PD target against *E. coli*. As Abbott et al. reported, the established ciprofloxacin target is AUC_0–24_/MIC of 878 for 1-log_10_ kill during 72 h in an *in vitro* bladder infection model (9). For the treatment of gram-negative bacterial infections, including *E. coli*, the PK/PD target that levofloxacin needs to achieve is AUC_0–24_/MIC of 125 (8, 27–29). Overall, the urinary PK/PD target that nemonoxacin should attain is rather lower than those of ciprofloxacin and levofloxacin.

When MICs were determined in SHU, the corresponding AUC_0–24_/MIC_SHU_ targets were approximately fourfold higher than AUC_0–24_/MIC, reflecting reduced activity in the urine matrix—also evident from the two to five twofold MIC increases in SHU versus CAMHB. While MIC_SHU_ may better reflect site-of-infection activity, its non-standardized nature limits clinical translation. The MIC should be measured by the reference method to provide a phenotypic endpoint in a defined and standardized system as a reproducible measure of antibiotic activity against that microorganism (30).

### Dosing regimen recommendation

In CAMHB-based analyses, MCS results support the *in vitro* efficacy of standard-dose nemonoxacin (500 mg orally daily) against *E. coli* with MIC ≤ 32 mg/L for a 1-log_10_ kill and MIC ≤ 16 mg/L for EC_90_. The 750 mg regimen yields similar PTA and CFR, suggesting a probable plateau between 500 and 750 mg. In contrast, SHU-based targets projected lower efficacy: 500 mg regimen achieved 1-log_10_ kill only at MIC ≤ 8 mg/L and EC_90_ only at MIC ≤ 2 mg/L, with CFRs of 80.5% and 59.36%, respectively; the 750 mg regimen performed similarly.

Bacteria in the CAMHB and SHU groups were exposed to different growth environments due to the distinct culture media used. Antimicrobial susceptibility testing and growth curves showed that bacteria grown in SHU exhibited higher MICs and enhanced resistance, for possible reasons discussed previously. Consequently, isolates cultured in CAMHB demonstrated greater susceptibility and higher PTA under the same exposure levels. These results emphasize that the target-site environment can significantly affect drug activity and bacterial response and should therefore be considered when evaluating dosing regimens. Given these divergent projections, an optimal regimen cannot be recommended solely from *in vitro* data. Fortunately, an ongoing Phase III clinical trial (registration number CTR20231866) should provide more evidence for nemonoxacin efficacy in uUTI patients and afford an opportunity to decide the PK/PD target according to clinical outcomes.

### Nemonoxacin’s potential advantages in the treatment of uUTIs

Oral antimicrobials, nitrofurantoin and fosfomycin, remain first-line agents for uUTIs, but due to insufficient blood concentrations, they are mainly used for the treatment of lower uUTIs (31, 32). In contrast, nemonoxacin achieves high concentrations in both blood and urine, suggesting potential utility for the treatment of both upper and lower uUTIs. Recently, there have been reports of decreased sensitivity to nitrofurantoin and fosfomycin in *E. coli* clinical isolates (33–35). However, there have been no resistance cases reported for nemonoxacin, supporting its further evaluation in uUTIs.

### Limitations

There are several limitations to our *in vitro* PK/PD study. First, the AUBKC endpoints and PD target endpoints (stasis vs 1-log_10_ CFU reduction) have not been clinically validated using outcomes data in uUTI patients. Therefore, the *in vitro* PK/PD targets should be interpreted with caution. Second, the PK/PD setting is simplified and does not consider bacterial dilution during bladder filling or loss during voiding (36). A more complex bladder infection *in vitro* model, as described by Abbott et al. (37), may better capture these processes; however, there are too many compartments in the whole setting, making it challenging to simulate nemonoxacin urine exposure perfectly with limited data. Third, urine is a complex biomatrix containing various components, such as components of the immune system (38) and sex-associated differences in urinary metabolites (39). In our study, pooled human urine samples were obtained from individuals of the same gender. Although certain urinary components may differ by sex, urine pH did not significantly differ between male and female subjects (6.193 vs 6.178, *P* = 0.6475). The SHU reflects key urine features but cannot capture its full biological complexity. Finally, our simulations used PK data from healthy volunteers, which may differ from patient populations.

### Conclusion

In summary, an *in vitro* UTI model directly replicates nemonoxacin urinary exposures to define its exposure–response relationships at the infection site. AUC_0–24_/MIC is the most predictive PK/PD index. In CAMHB, the AUC_0–24_/MIC target for a 1-log_10_ CFU reduction from baseline was 46.5, and MCS results support efficacy of the standard oral dose (500 mg once daily) against *E. coli* isolates with MIC ≤ 16 mg/L. Yet, these findings still require confirmation in clinical studies.

## Data Availability

The data that support the findings of this study are available from the corresponding author and the sponsor upon reasonable request.

## SUPPLEMENTAL MATERIAL

Supplemental material aac.01368-25-s0001.docx Tables S1 to S3.

ASM does not own the copyrights to Supplemental Material that may be linked to, or accessed through, an article. The authors have granted ASM a non-exclusive, world-wide license to publish the Supplemental Material files. Please contact the corresponding author directly for reuse.
