# Supplementary material for: Nemonoxacin pharmacokinetics/pharmacodynamics against Escherichia coli in an in vitro dynamic urinary tract infection model
Source: Antimicrob Agents Chemother. 2026 Jun 11;70(7):e01368-25. doi: 10.1128/aac.01368-25 (PMC13321827; doi:10.1128/aac.01368-25)
Supplement: Supplemental material — Tables S1 to S3. [file aac.01368-25-s0001.docx]

**Supplemental Materials**

Nemonoxacin Pharmacokinetics/pharmacodynamics against *Escherichia coli* in an *in vitro* Dynamic Urinary Tract Infection Model

Chunye Qi, Ruohao Zhang, Xingyi Qu, Xiaofen Liu, Xingchen Bian, Fengjia Zhu, Jing Chen, Xin Li, Jing Zhang

**Table Captions**

**Table S1** Chemicals and their concentrations in the SHU media recipe for studies of infection.

**Table S2** Flow rates of the antibiotic solution (in the absorption phase) and the drug-free medium (in the elimination phase) for the high exposure and low exposure group, respectively, over different time intervals.

**Table S3** MIC values of nemonoxacin and levofloxacin against ESBL+ and ESBL- *E. coli* cultured in CAMHB and SHU.

**Table S1**  Chemicals and their concentrations in the SHU media recipe for studies of infection.

| **Chemical^a^** | | **Concentration (mM)** | **Amount per 1L (g)** |
| --- | --- | --- | --- |
| Sodium chloride | NaCl | 100 | 5.844 |
| Sodium sulphate | Na_2_SO_4_ | 17.0 | 2.4147 |
| Urea | Urea | 280 | 16.8168 |
| Potassium chloride | KCl | 38.0 | 2.8329 |
| Calcium chloride | CaCl_2_ | 4.0 | 0.4439 |
| Creatinine | Creatinine | 9.0 | 1.0181 |
| Citric acid trisodium salt dihydrate | Na_3_C_6_H_5_O_7_ | 3.4 | 0.9999 |
| Ammonium chloride | NH_4_Cl | 20.0 | 1.0698 |
| Magnesium sulphate | MgSO_4_ | 3.2 | 0.3852 |
| Sodium oxalate | Na_2_C_2_O_4_ | 0.18 | 0.0241 |
| Sodium phosphate monobasic | NaH_2_PO_4_ | 3.6 | 0.5616 |
| Sodium phosphate dibasic | Na_2_HPO_4_ | 6.5 | 0.9227 |
| Potassium dihydrogen phosphate | KH_2_PO_4_ | 16.0 | 2.1774 |
| Uric acid | C_5_H_4_N_4_O_3_ | 0.6 | 0.1009 |
| Sodium bicarbonate | NaHCO_3_ | 13.5 | 1.1341 |
| Magnesium chloride hexahydrate | MgCl_2_ | 3.2 | 0.6506 |
| Lactic acid | C_3_H_6_O_3_ | 1.1 | 0.0991 |
| Ferrous sulphate heptahydrate | FeSO_4_·7H_2_O | 0.005 | 0.0014 |
| 20% (w/v) casamino acids | - | - | 0.1%(v/v) |
| 10% (w/v) yeast extract | - | - | 0.1%(v/v) |

^a^ All chemical materials were bought from Sangon Biotech, China.

**Table S2** Flow rates of the antibiotic solution (in the absorption phase) and the drug-free medium (in the elimination phase) for the high exposure and low exposure group, respectively, over different time intervals.

|  | **Low exposure** | | **High exposure** | |
| --- | --- | --- | --- | --- |
|  | Time interval  (min) | Flow rate  (mL/min) | Time interval  (min) | Flow rate  (mL/min) |
| Absorption phase | 0-30 | 2.38 | 0-30 | 1.09 |
|  | 30-45 | 1.55 | 30-54 | 0.41 |
|  | 45-58 | 1.37 | 54-63 | 0.20 |
|  | 58-63 | 0.96 | / | / |
|  | 63-84 | 0.44 | / | / |
|  | / | / | / | / |
| Elimination phase | 84-210 | 0.24 | 63-120 | 0.15 |
|  | 210-330 | 0.23 | 120-240 | 0.23 |
|  | 330-450 | 0.10 | 240-360 | 0.09 |
|  | 450-600 | 0.07 | 360-1440 | 0.03 |
|  | 600-1440 | 0.06 | / | / |

**Table S3** MIC values of nemonoxacin and levofloxacin against ESBL+ and ESBL- *E. coli* cultured in CAMHB and SHU.

| **Isolates** | **Type** | **CAMHB** | | **SHU-pH5.6** | | **SHU-pH6.0** | | **SHU-pH6.5** | |
| --- | --- | --- | --- | --- | --- | --- | --- | --- | --- |
|  |  | **NEN** | **LEF** | **NEN** | **LEF** | **NEN** | **LEF** | **NEN** | **LEF** |
| 21-W031-023 | ESBL+ | 4 | 2 | 64 | 32 | 32 | / | 16 | 8 |
| 21-W033-001 | ESBL+ | 0.5 | 0.5 | 64 | 4 | 8 |  | 2 | 4 |
| 21-W035-079 | ESBL+ | 8 | 16 | 256 | 128 | 32 |  | 32 | 64 |
| 21-W049-099 | ESBL+ | 32 | 16 | 256 | 128 | 256 |  | 128 | 16 |
| 21-W061-049 | ESBL+ | 2 | 2 | 32 | 32 | 64 |  | 16 | 4 |
| 21-W031-028 | ESBL- | 1 | 0.5 | 64 | 8 | 16 |  | 4 | 1 |
| 21-W041-052 | ESBL- | 1 | 0.5 | 64 | 16 | 16 |  | 2 | 0.5 |
| 21-W042-007 | ESBL- | 16 | 8 | 256 | 64 | >512 |  | 64 | 32 |
| 21-W049-080 | ESBL- | 2 | 1 | 64 | 8 | 16 |  | 8 | 1 |
| 21-W049-027 | ESBL- | 16 | 8 | 512 | 128 | 256 |  | 64 | 32 |
| ATCC 25922 | - | <0.03 | <0.125 | 0.5 | 2 | 0.5 |  | 0.5 | <0.5 |
| NTU-001 | / | 1 | / | / | | / |  | 64 | / |
| NTU-002 | / | 128 |  |  |  |  |  | 512 |  |
